# Induced defence by a root hemiparasite increases host plant resistance against future infection

**DOI:** 10.1111/plb.70187

**Published:** 2026-02-01

**Authors:** S. C. Wanke, D. Matthies

**Affiliations:** ^1^ Plant Ecology, Department of Biology Philipps‐Universität Marburg Marburg Germany

**Keywords:** Hemiparasite–host interactions, host suppression, *Melampyrum arvense*, parasite survival, *Rhinanthus alectorolophus*

## Abstract

- European grassland plants are frequently attacked by root hemiparasites. However, little is known about host defence responses to parasitism. We investigated whether prior parasitization by a root hemiparasite makes hosts more susceptible to parasitism or, on the contrary, stimulates host defence against a future attack by hemiparasites.
- We grew three host species (*Lolium perenne*, *Trifolium repens* and *Sanguisorba minor*) in phase 1 for 3 months with the hemiparasite *Rhinanthus alectorolophus,* removed the parasite and grew the same host individuals in phase 2 with the hemiparasites *R. alectorolophus* or *Melampyrum arvense.*
- Previous infection by a parasite reduced the survival of the seedlings of *Rhinanthus* and *Melampyrum* with all host species but increased the biomass of the surviving parasites. A previous infection reduced the biomass of the hosts in most treatment combinations, but variation in initial host biomass at the start of phase 2 only partly explained this effect. Some of these interactions were specific to particular parasite–host species combinations.
- The results indicate that infection by root hemiparasites induces in the hosts defence mechanisms against future infection by the parasites (increased pre‐attachment resistance), but parasite individuals that overcome this defence may then also be particularly good at exploiting the hosts (no increased post‐attachment resistance). Thus, infection by root hemiparasites may activate host defence pathways that can influence future interactions with herbivores and pathogens and thus community dynamics.

## Graphical abstract

Infection by root hemiparasites can increase mortality of parasites during future infection, indicating induced host defence mechanisms.

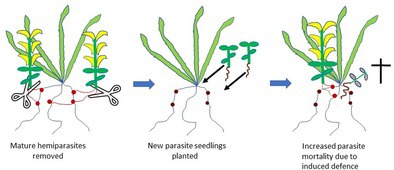

## INTRODUCTION

One of the many antagonistic interactions plants face is parasitism by hemi‐ and holoparasitic plants. There are c. 4750 plant species known to parasitize other plants (Těšitel 2016; Nickrent 2020). Parasitic plants attach to the stems or roots of their host plants via the formation of haustoria, that is specialized organs that form vascular connections between host and parasite, and extract water and solutes from their hosts (Yoshida *et al*. 2016). Holoparasites rely completely on their hosts for obtaining carbon, while hemiparasites have green leaves and are able to photosynthesize. All holoparasites are obligate parasites, while some root hemiparasites like *Rhinanthus* ssp., *Euphrasia* ssp. and *Odontites* ssp. are facultative parasites and are able to complete their life cycle without a host, although they remain rather small (Seel *et al*. 1993; Matthies 1998). Obligate parasites are not able to survive if they do not successfully attach to a host, but even facultative parasites often die because of competition by the surrounding vegetation (Fibich *et al*. 2010; Těšitel *et al*. 2015). Some parasitic plant species like *Striga* ssp. and *Orobanche* ssp. cause immense agricultural losses, especially in countries where sophisticated control measures are not widely available and pose a serious threat to global food security (Parker 2012; Gressel & Joel 2013). In Europe, the root hemiparasites *Melampyrum arvense* and *Rhinanthus* ssp. were formerly considered serious weeds of crops (Zając & Zając 2014; Matthies 2017), while today they are restricted to grasslands.

The benefits for hemiparasites of attachment to a host vary greatly among different host species (Hautier *et al*. 2010; Matthies 2017, 2021). Host quality is to a large extent determined by specific interactions between individual host and parasite species, but there is also a significant phylogenetic component in the quality of species as hosts for hemiparasite (Brown *et al*. 2021; Moncalvillo *et al*. 2025). Root hemiparasites act as an additional sink for the resources taken up by the roots of the hosts, as well as carbon (Těšitel *et al*. 2010). This results in reduced growth of the host (Ameloot *et al*. 2005; Phoenix & Press 2005) and changes in patterns of biomass allocation (Matthies 1998, 2017, 2021; Puustinen & Salonen 1999). Since parasites have high transpiration rates, even at night (Press & Phoenix 2005), hosts often activate their drought‐stress responses and close their stomata to limit transpiration, thereby decreasing stomatal conductance and photosynthesis (Watling & Press 2001). To compensate for the loss of water and nutrients to the parasite, hosts allocate more of their biomass to root growth, which leads to a higher root mass fraction (Graves 1995; see review in Matthies 2017).

Plants can show varying degrees of resistance against parasitic plants, both before (‘pre‐attachment resistance’) and after parasite attachment (‘post‐attachment resistance’; Clarke *et al*. 2019; Jhu & Sinha 2022). Host resistance mechanisms can prevent the parasite from forming functional connections with the host or hinder parasite development (Albert *et al*. 2021). Pre‐attachment resistance mechanisms include the release of compounds inhibiting parasite germination or seedling development and reduced haustorium initiation and invasion (Serghini *et al*. 2001; Echevarría‐Zomeño *et al*. 2006; Fishman & Shirasu 2021). Mechanisms of post‐attachment resistance include the following (Timko & Scholes 2013; Albert *et al*. 2021): (1) Synthesis and release of cytotoxic compounds such as phenolic acids and phytoalexins leading to decreased vitality or even death of the infected plant organ (Olivier *et al*. 1991; Jorrín *et al*. 1996; Hood *et al*. 1998); (2) prevention of pathogen ingress and growth through the formation of physical barriers by lignification and/or callose deposition (Irving & Cameron 2009); and (3) the induction of a hypersensitive response which includes the release of reactive oxygen species and programmed cell death to limit parasite development (Timko & Scholes 2013).

These pre‐ and post‐attachment resistance mechanisms are part of the innate immune system of plants. Unlike vertebrates, plants do not possess an adaptive immune system since they lack the circulatory system and specialized immune cells required for it (Spoel & Dong 2012). They do, however, have immunologic memory (Spoel & Dong 2012; Reimer‐Michalski & Conrath 2016), which has been observed in multiple cases of infection by bacteria, fungi and viruses (Kuć 1982), and herbivory (Reimer‐Michalski & Conrath 2016). Immune memory requires the induction of a systemic immune response after local infection or tissue damage (Spoel & Dong 2012). In a process known as ‘cell priming’, non‐infected tissues are put into a state of heightened sensitivity, which enables them to react more quickly and with greater force to future infection or wounding (Conrath *et al*. 2006).

While there is a large volume of research on plant defences against herbivores, little is known about induced defences against parasitic plants (Pennings & Callaway 2002). Previous infection of plants by parasites has been found to slightly slow the attachment of the shoot parasite *Cuscuta* to tomato plants, while prior herbivory did not (Tjiurutue *et al*. 2016). This suggests that parasitism may induce a specific immunological memory. By contrast, in a study with the root hemiparasite *Rhinanthus minor*, parasites grown on *Poa alpina* that had been parasitized in the previous year accumulated twice as much biomass as those growing on hosts infected for the first time (Seel & Press 1996). In this case, previous parasitism may have stressed the hosts and made them more susceptible to future infections, similar to increased susceptibility to pathogen infection after experience of abiotic stresses (Suzuki *et al*. 2014; Ramegowda & Senthil‐Kumar 2015). The aim of this study was to assess whether parasitization by a root hemiparasite may stimulate host defence against future attack by hemiparasites or make hosts more susceptible (Pennings & Callaway 2002).

We studied the effect of prior experience of parasitism by the root hemiparasite *Rhinanthus alectorolophus* (Scop.). Pollich on the interactions between three host species (*Lolium perenne* L., *Trifolium repens* L. and *Sanguisorba minor* Scop.) and the root hemiparasites *R. alectorolophus* and *M. arvense* L. We asked the following specific questions: (1) Is survival and growth of the parasites increased or reduced if the host had been parasitized previously? (2) Do the effects of prior parasitization vary among hosts and parasite species?

## MATERIALS AND METHODS

### Study species

The two root hemiparasites *Rhinanthus alectorolophus* (Scop.) Pollich and *Melampyrum arvense* L. (both Orobanchaceae) used to be common in central Europe but have much declined in recent decades due to the intensification of agriculture (Blažek & Lepš 2015; Matthies 2017). *M. arvense* was formerly a weed of cereal crops but is nowadays restricted to various types of grasslands (Matthies 2017). *R. alectorolophus* is a grassland species, but an ecotype of the species (subsp. buccalis) was formerly a weed of cereals (Zając & Zając 2014). The two parasite species do not depend on specific hosts, but their performance strongly varies with different host species (Matthies 2017, 2021). Three perennial species representing different functional groups were used as hosts: the grass *Lolium perenne*, the forb *Sanguisorba minor* and the legume *Trifolium repens*. The species were chosen because they are good hosts for the studied parasites and occur in central European grasslands together with them (Matthies 2017, 2021).

### First phase of the experiment

**Fig. 1:**
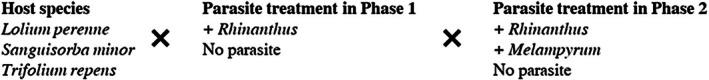
Experimental design of the two‐phase study of the effects of prior parasitism by *Rhinanthus alectorolophus* on the interactions between three host species (*Lolium perenne, Sanguisorba minor, Trifolium repens*) and two hemiparasites (*R. alectorolophus* and *Melampyrum arvense*).

The experiment consisted of two phases. In phase 1, the three host species were grown with and without the hemiparasite *R. alectorolophus*. The parasites were then removed and in phase 2, the same host individuals were grown with the parasites *R. alectorolophus* or *M. arvense*, while one set of hosts served as a control (Fig. 1).

Seeds of the parasites and the hosts were acquired from a commercial supplier (Appels Wilde Samen, Darmstadt, Germany). In mid‐August 2021, seeds of *Rhinanthus* were placed in Petri dishes on moist filter paper and incubated at 5 °C until cotyledons developed. The seeds of the hosts were germinated in late October 2021 on moistened filter paper at room temperature. The host seedlings were transplanted individually into 9 × 9 × 9.5 cm pots filled with a 2:1 mixture of sand and peat substrate (HAWITA^®^ professional, Vechta, Germany), and 2 ml of local grassland soil were added to each pot to inoculate the soil with microbia. For each host, 60 replicate pots were set up. The pots were placed individually on saucers and kept in a growth chamber at 22 °C, 60% relative humidity and a 10 h/14 h day/night cycle. Lighting was by metal halide lamps (c. 220 μmol photons m^−2^ s^−1^ at pot level). After 3 weeks of growth, three seedlings of *Rhinanthus* were planted into 30 pots of each host treatment. Seedlings that died during the first 2 weeks were replaced. Every 2 days the plants were watered until the soil was saturated. The position of the pots was randomized every 2 weeks. After 68 days of growth, the parasites were harvested above ground and killed by cutting them at ground level. The harvested material was dried at 80 °C for 48 h and weighed. To obtain non‐destructive estimates of host size, for *L. perenne* the number of shoots per plant was counted and the maximum length of a leaf was determined, for *S. minor* the number of leaves, the width of the largest terminal leaflet and the diameter of the shoot at ground level and for *T. repens* the number of leaves and the length and width of the largest leaflet.

### Estimation of initial host biomass in phase 2 from non‐destructive measurements

In addition to the experimental plants, another 44 plants of each host species were set up and cultivated under the same conditions. These plants were harvested above and below ground at different ages, and several traits related to size were determined to obtain regression equations for predicting total host biomass from non‐destructive measurements. For *L. perenne*, the number of shoots and maximum leaf length were measured, but only the number of shoots proved to be a good predictor of biomass: Log biomass of *L. perenne* (g) = −0.6917 + 0.07367 × (number of shoots); r^2^ = 0.78, *P* < 0.001. For *T. repens*, the length and width of the largest central leaflet and the number of leaves were recorded, but only the number of leaves was a good predictor of biomass: Biomass of *T. repens* (g) = −0.2793 + 0.1070 × number of leaves; r^2^ = 0.70, *P* < 0.001. For *S. minor*, the width of the largest terminal leaflet, the number of leaves and shoot diameter were recorded. The best predictive equation was: Biomass of *S. minor* (g) = 3.2264 + 1.3905 × shoot diameter [mm] − 2.5600 × leaflet width [cm]; r^2^ = 0.82, *P* < 0.001. Based on these equations, the initial mass of each host at the start of phase 2 of the experiment was estimated.

### Second phase of the experiment

Seeds of the parasites for the second phase of the experiment (*Rhinanthus* and *Melampyrum*) were set up in November 2021 on moist filter paper in Petri dishes at 5 °C. Ten pots per combination of host species and *Rhinanthus* treatment during phase 1 of the experiment received each two seedlings of *Rhinanthus* on 7 February 2022 and a second set of 20 pots served as control without parasites. Because germination of the *Melampyrum* seeds took longer and was lower than expected, the number of replicates for the *Melampyrum* treatment had to be reduced and two seedlings of *Melampyrum* were planted into a set of only five pots per treatment combination on 16.3.2022. Parasite seedlings that died were counted and replaced for 3 weeks. All pots received 50 ml of a 2 gl^−1^ solution of fertilizer (Hakaphos® Gartenprofi; NPK 14:7:14%; Compo, Münster, Germany) on 24 February and 10 March 2022. On 12 and 13 May 2022, the number of surviving parasites per pot was counted, and parasites and hosts were separately harvested above ground, dried for 48 h at 80 °C and weighed. The pots containing the roots were immediately frozen at −20 °C for later analysis. The pots were then successively thawed by submerging them in hot water, and the roots were washed free of soil. Because the roots of small parasites were difficult to identify, parasite roots were removed as far as possible and only the roots of the hosts dried and weighed.

### Statistical analyses

All analyses were conducted using R 4.4.2 (R Core Team 2024). All statistical models were successively simplified by removing terms until a model with minimum AIC was obtained. The effects of host species and the number of surviving parasites on mean parasite biomass in phase 1 were studied by analysis of covariance, and the effects of host species and parasitism by *Rhinanthus* on estimated above‐ground host biomass and productivity per pot (host + parasite) were examined by two‐way analyses of variance.

The effects of host species, prior parasitism of the hosts by *Rhinanthus* in phase 1 and parasite species (*R. alectorolophus* or *M. arvense*) in phase 2 on parasite survival were analysed by a generalized linear model with a logit link and a binomial error distribution (function *glm*) and Wald chi^2^‐tests (function ANOVA, *car*‐package, Fox & Weisberg 2019). To adjust for initial differences in host size, a further analysis that included estimated host biomass at the start of phase 2 as a covariate was conducted.

The effect of host species identity, prior parasitism and parasite species (*R. alectorolophus* or *M. arvense*) on mean above‐ground biomass of the surviving parasites within a pot in phase 2 was analysed by 3‐factorial ANOVA. Additional analyses tested separately the effects of initial host biomass and the number of parasites per pot as covariates. Similar analyses were carried out for effects on host biomass, productivity per pot and the proportion of biomass allocated to roots (RMF), but with the factor parasite treatment with three levels (+*R. alectorolophus*, +*M. arvense*, no parasite).

To obtain normally distributed residuals, all biomass data were log‐transformed prior to analysis. Marginal means were obtained with the package *emmeans* (Lenth 2022). Simple main effects analyses were carried out using marginal means and the *test(pairs)* command. Box plots of raw data for measured variables are shown as Supporting information S1.

## RESULTS

### Parasite and host performance in phase 1

**Fig. 2:**
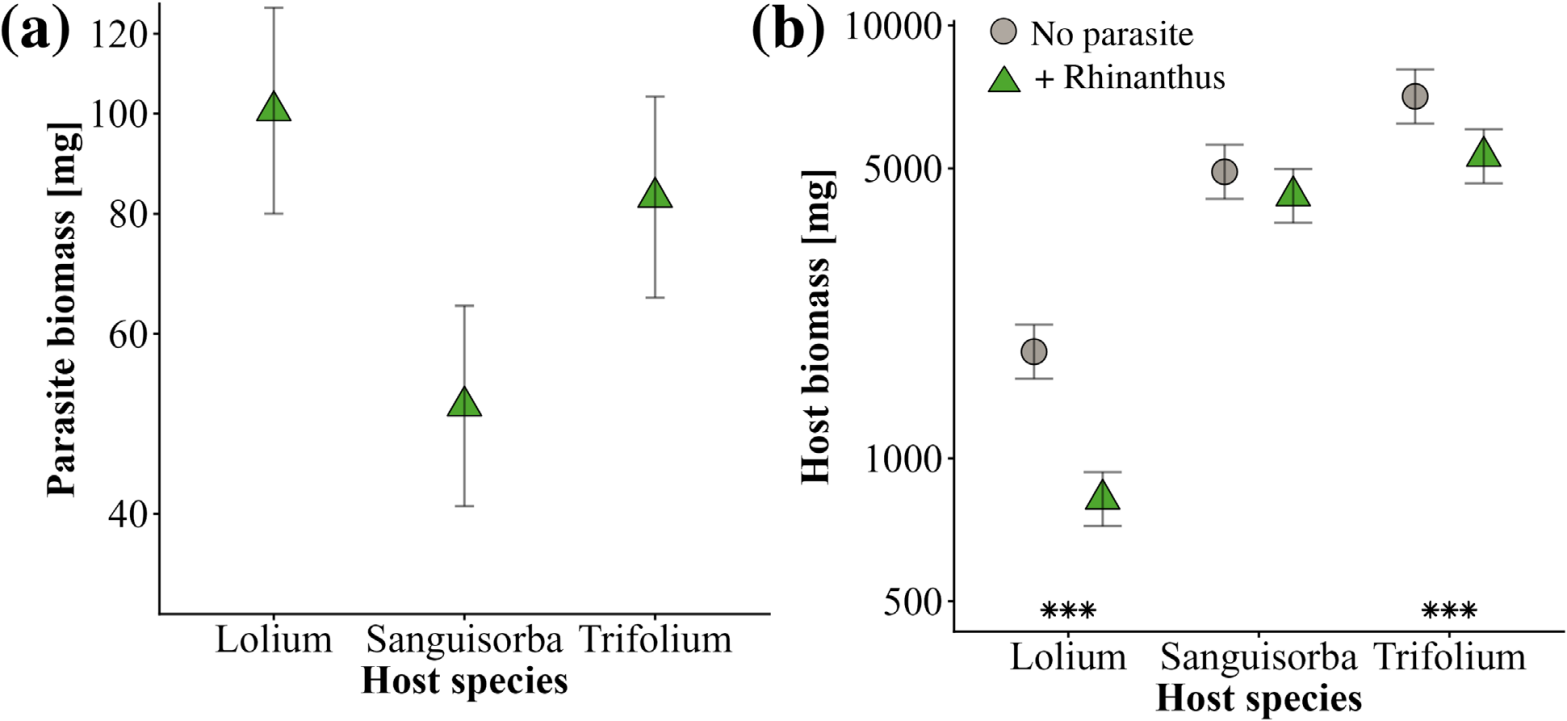
Mean biomass of (a) the parasite *Rhinanthus alectorolophus* grown with three different host species (*Lolium perenne, Sanguisorba minor, Trifolium repens*) adjusted for the effect of the number of surviving parasites, and (b) estimated biomass of the host species when grown with and without *R. alectorolophus* at the end of phase 1 of the experiment. Means ±95% confidence intervals. ***P < 0.001 for simple main effects of the presence of *R. alectorolophus* within host treatments.

**Fig. 3:**
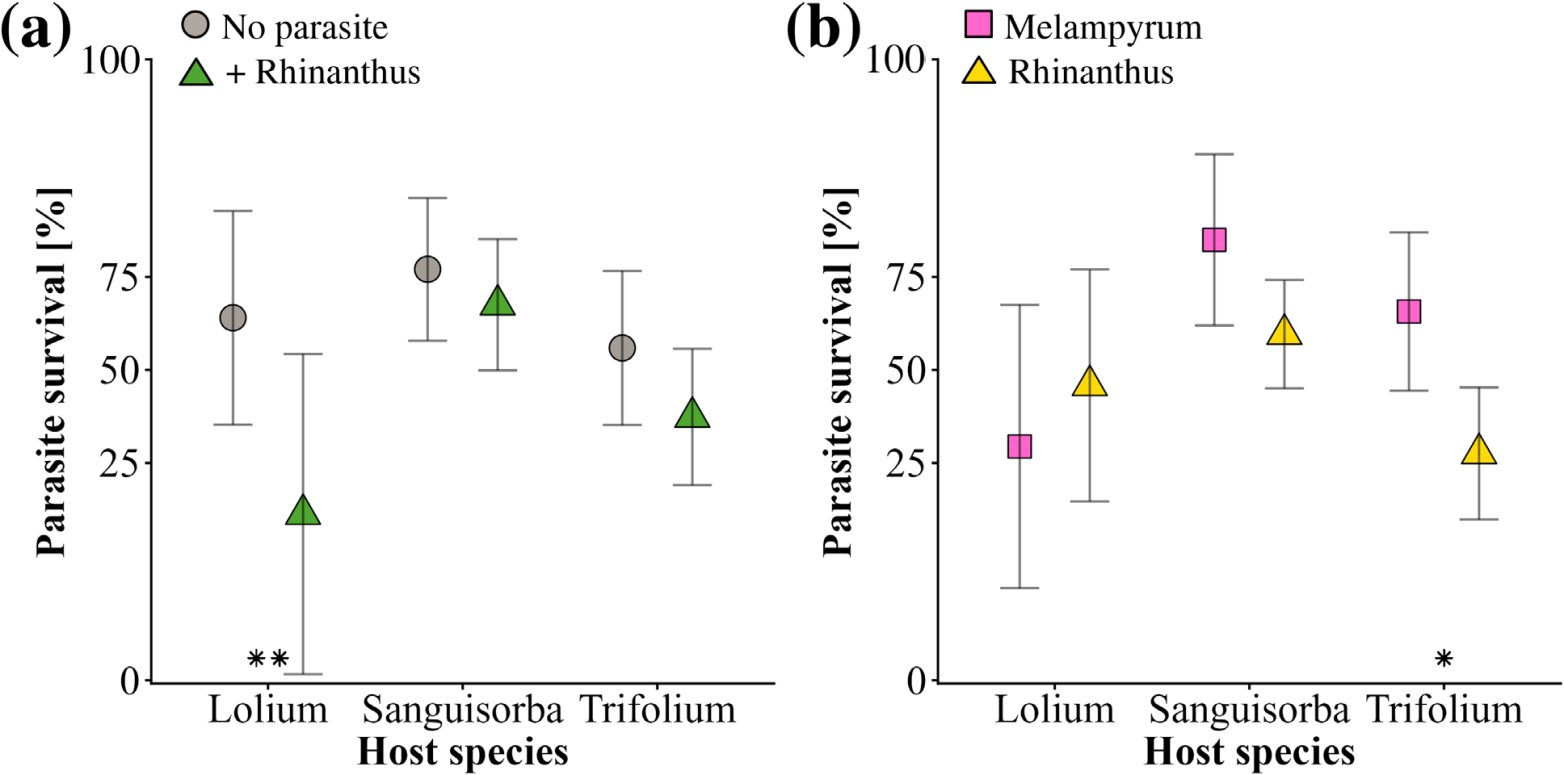
(a) Effect of parasitism of the hosts by *Rhinanthus alectorolophus* during phase 1 on survival of the parasites *Melampyrum arvense* and *R. alectorolophus* in phase 2, and (b) differences in survival between *M. arvense* and *R. alectorolophus*. The parasites were grown with three different host species (*Lolium perenne, Sanguisorba minor, Trifolium repens*). Means ±95% confidence intervals. Effects were adjusted for the effects of initial host biomass at the start of phase 2. *P < 0.05; **P < 0.01 for simple main effects of parasitism within host treatments.

The mean total biomass of the parasites in a pot at the end of phase 1 varied depending on the host species and the number of parasites per pot (Table 1). Parasite biomass was lower with *S. minor* than with *L. perenne* and *T. repens* (Fig. 2). Each additional parasite in a pot reduced the mean biomass per parasite by 19%. Both parasitism and host species identity influenced host biomass, and the suppression of host biomass by the parasite *R. alectorolophus* varied among host species. The biomass of *L. perenne* was most strongly affected by parasitism (−54%, Fig. 3), while *T. repens* (−28%) and *S. minor* (−12%) were suppressed less. Total above‐ground productivity (host + parasite) was also reduced by the parasite (−14%), but this effect hardly varied among host species (F_2,144_ = 0.84, *P* = 0.435).

**Table 1:** Analyses of variance of the effects of the number of parasites and host species (*Lolium perenne, Sanguisorba minor, Trifolium repens*) on the above‐ground biomass of the hemiparasite *Rhinanthus alectorolophus,* and of the effects of parasitism on the estimated total biomass of the hosts and on total productivity (host + parasite). Residual df = 71 for parasite biomass, df = 144 for host biomass and df = 146 for productivity. Models are presented for which the Akaike information criterion is minimal.

source of variation	df	parasite biomass		host biomass		productivity	
		F	*P*	F	*P*	F	*P*
Number of parasites	1	9.45	0.003				
Host species	2	8.83	<0.001	269.36	<0.001	258.38	<0.001
Parasitism	1			47.71	<0.001	8.24	0.005
Host species × parasitism	2			10.76	<0.001		

### Parasite survival and growth in phase 2

Parasite survival in phase 2 varied among host species and parasite species and was influenced by whether the host had experienced parasitism during phase 1 (Table 2). Survival of parasites grown with host plants that had already experienced parasitism in phase 1 was significantly lower than that of control plants (53% *versus* 67%), and there was some evidence that the survival of parasites depended on the combination of parasite and host species. Parasite survival decreased with increasing host biomass at the time of parasite planting. When the analysis was adjusted for variation in initial host biomass, the effects of prior parasitism by *Rhinanthus* and the differences in survival of the two parasite species became more pronounced (Table 2). Mean survival of parasites grown with naive hosts was 67%, while only 38% survived grown with hosts that had experienced parasitism in phase 1. There was also some evidence that the effects of prior parasitism depended on the host species, with parasites grown with *L. perenne* particularly strongly affected (Fig. 3a). Effects of prior parasitism of the hosts on parasite survival were much weaker for parasites grown with *T. repens*, and there was hardly any effect on those grown with *S. minor*. Survival of the parasites also varied between the two parasite species, in particular when grown with *T. repens*. With *T. repens* as host, survival was much higher for *M. arvense* than for *R. alectorolophus* (Fig. 3b).

**Table 2:** Analyses of deviance (Wald chi‐squared tests) of the effects of host biomass, host species (*L* *olium perenne, Sanguisorba minor, Trifolium repens*), prior parasitism by *Rhinanthus alectorolophus* and parasite species on survival probability of the hemiparasites *R. alectorolophus* and *Melampyrum arvense*. Analyses were carried out without (df_Res_ = 83) and with initial host mass as a covariable (df_Res_ = 80). Models are presented for which the Akaike information criterion is minimal.

source of variation	df	χ^2^	*P*	χ^2^	*P*
Log10 (initial host mass)	1			5.51	0.019
Host species	2	24.72	<0.001	12.19	0.002
Prior parasitism	1	4.19	0.041	6.48	0.011
Parasite species	1	4.11	0.043	5.04	0.025
Prior parasitism × host species	2			4.95	0.084
Parasite species × host species	2	5.52	0.063	8.62	0.013

**Fig. 4:**
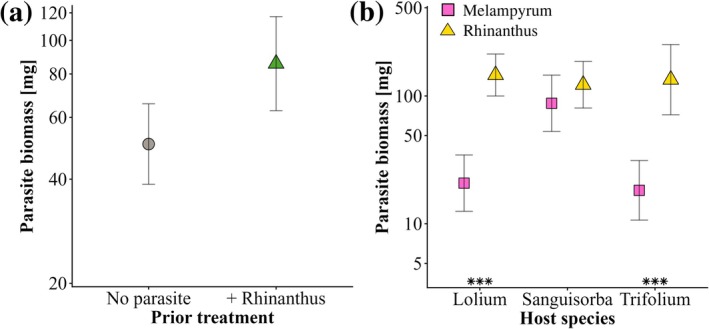
(a) Effect of parasitism of the hosts by *Rhinanthus alectorolophus* during phase 1 on the mean above‐ground biomass of the parasites *R. alectorolophus* and *Melampyrum arvense* at the end of phase 2, and (b) mean biomass of the parasites *R. alectorolophus* and *M. arvense* grown with three host species (*Lolium perenne, Sanguisorba minor, Trifolium repens*) at the end of phase 2. Means ±95% confidence intervals. ***P < 0.001 for simple main effects of parasite species within host treatments.

Parasite biomass at the end of phase 2 was also influenced by prior parasitism of the hosts (Table 3). Overall, the mean biomass of the parasites was 70% higher if the hosts had been parasitized during phase 1 (Fig. 4a). The biomass of *M. arvense* was much lower than that of *R. alectorolophus* when grown with *L. perenne* or *T. repens*, but similar to that of *S. minor* (Fig. 4b). The host biomass at the start of phase 2 had little effect on mean parasite biomass (F_1,59_ = 1.15, *P* = 0.289), and there was also little evidence that the number of parasites influenced their mean biomass (F_1,59_ = 1.60, *P* = 0.210).

**Table 3:** Analysis of variance of the effects of host species (*Lolium perenne, Sanguisorba minor, Trifolium repens*), prior parasitism of the host and parasite species (*Rhinanthus alectorolophus* or *Melampyrum arvense*) on the mean above‐ground biomass of the parasites at the end of phase 2. The model is presented for which the Akaike information criterion is minimal.

source of variation	df	F	*P*
Host species	2	5.30	0.008
Prior parasitism	1	6.85	0.011
Parasite species	1	48.58	<0.001
Prior parasitism × host species	2	2.21	0.119
Parasite species × host species	2	7.78	<0.001
Residual	60		

### Host performance in phase 2

**Fig. 5:**
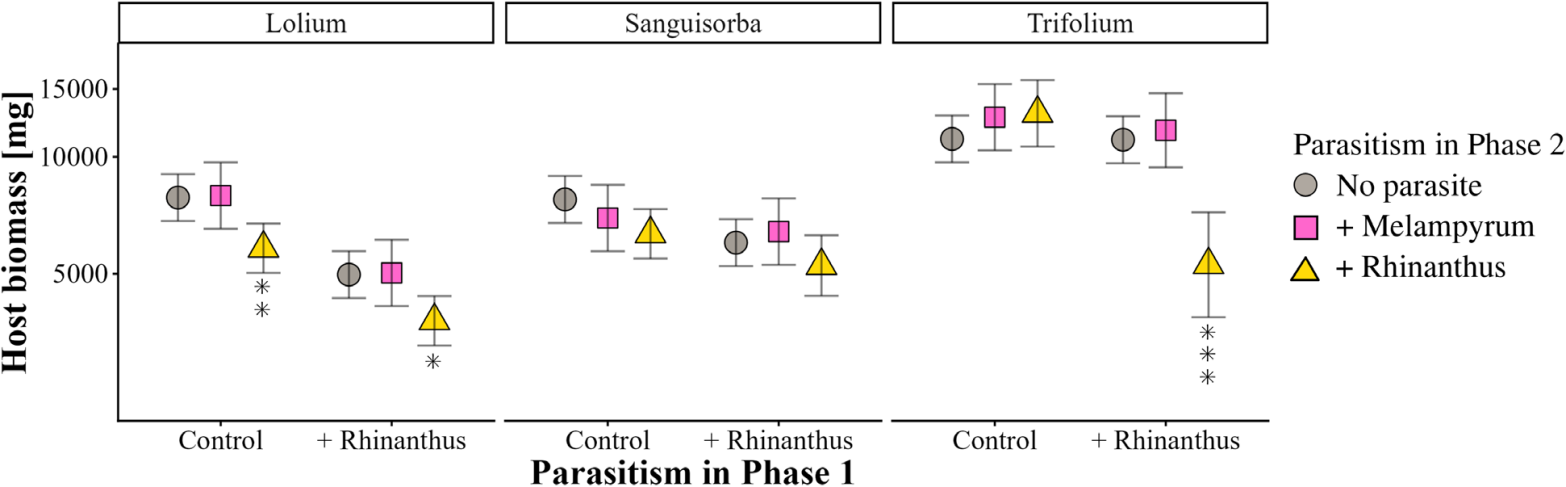
Effects of prior parasitism by *Rhinanthus alectorolophus* on the biomass of three different host species (*Lolium perenne, Sanguisorba minor, Trifolium repens*) parasitized by *Melampyrum arvense* or *R. alectorolophus*. Asterisks above groups indicate significant differences (*P < 0.05; **P < 0.01; ***P < 0.001) within each group calculated from simple main effects. Means ±95% confidence intervals.

The total biomass of the hosts (above and below ground) was influenced by host species identity, prior parasitism and parasite treatment in phase 2 (Table 4). Parasitism by *Rhinanthus* during phase 1 reduced host biomass at the end of phase 2 by on average 28% and parasitism by *Rhinanthus* in phase 2 reduced it by 23%, while parasitism by *Melampyrum* had on average no negative effect (+2.8%). However, the effect of prior parasitism and parasite treatment during phase 2 also depended on the host species (Fig. 5; 3‐way interaction in Table 4). The biomass of *L. perenne* was reduced by the parasite *Rhinanthus*, both if the host had been parasitized during phase 1 (−25%) and if not (−27%). By contrast, *Rhinanthus* only reduced the biomass of *T. repens* hosts that had experienced parasitism during phase 1 (−53%), while *Melampyrum* had no negative effect. *S. minor* was not clearly affected by either of the parasites.

**Table 4:** Analyses of variance of the effects of host species identity (*Lolium perenne, Sanguisorba minor, Trifolium repens*), prior parasitism by *Rhinanthus alectorolophus* during phase 1 and parasite treatment during phase 2 (*R. alectorolophus, Melampyrum arvense*, no parasite) on final host biomass. Analyses were carried out without (df_Res_ = 111) or with initial host biomass at the start of phase 2 as a covariate. Models are presented for which the Akaike information criterion is minimal.

source of variation	df	F	*P*	F	*P*
Initial host biomass	1			15.68	<0.001
Host species	2	63.32	<0.001	35.22	<0.001
Prior parasitism	1	54.04	<0.001	19.11	<0.001
Parasite treatment in phase 2	2	15.45	<0.001	9.38	<0.001
Prior parasitism × host species	2	4.03	0.020	0.81	0.446
Parasite treatment × host species	4	0.83	0.509	0.72	0.575
Prior parasitism × parasite treatment	2	4.43	0.014	3.02	0.053
Prior parasitism × parasite treatment × host species	4	4.18	0.003	2.36	0.058

Including initial host biomass as a covariate in the analysis of host biomass corrects for size differences among hosts developed during phase 1 and allows testing the effect of the factors of interest on the biomass gain of the hosts during phase 2. Effects became less pronounced, but parasitism during phase 1 still reduced biomass gain by 19%, indicating that the negative effect of prior parasitism on final host biomass was only partially due to a lower biomass at the beginning of phase 2. Total productivity (host + parasite) was strongly correlated with host biomass (r = 0.997), and the effects on productivity were similar, but less pronounced than those on host biomass: Prior parasitism reduced total productivity by 26% and the presence of *Rhinanthus* during phase 2 reduced it by 18%.

Of the experimental factors, only host species identity had a clear effect on their proportion of biomass allocated to roots (RMF; F_2,111_ = 79.8, *P* < 0.001), while there was little support for an effect of other factors or their interactions (all *P* > 0.39; except for host species × parasite treatment, *P* = 0.153). The RMF of *L. perenne* (51%) was higher than that of *S. minor* (41%), and the RMF of *T. repens* was lowest (21%).

## DISCUSSION

We investigated the effect of a prior infection by the root hemiparasite *R. alectorolophus* on the interactions between two root hemiparasites and three different host species. We hypothesized that the first infection would either induce host defences and decrease parasite performance during a second infection, or weaken the hosts and make them more susceptible to the parasites (Pennings & Callaway 2002). Survival of both *R. alectorolophus* and *M. arvense* was lower if the host plants had been parasitized previously. However, the parasites that survived when grown with previously parasitized hosts produced more biomass. This suggests that parasitism induced defence mechanisms which were more effective in preventing parasite attachment (pre‐attachment resistance) than in preventing resource extraction after attachment (post‐attachment resistance; Jhu & Sinha 2022).

Prior parasitism by *R. alectorolophus* during phase 1 reduced host biomass even in phase 2, but carry‐over effects from parasitism during phase 1 only partially explained this effect. This additional reduction in biomass may be due to a trade‐off between increased parasite resistance and growth, as has been observed in cases of plant resistance to pathogen infection (Walters & Heil 2007).

### Parasite–host interactions during phase 1 of the experiment

*Rhinanthus alectorolophus* successfully parasitized all three studied host species, but in line with the results of other studies, different species varied in their quality as hosts for the parasite and in the degree to which they were suppressed by parasitism (Rowntree *et al*. 2014; Matthies 2017, 2021; Moncalvillo & Matthies 2023). *L. perenne* was by far the best host and was damaged most strongly, followed by *T. repens* and *S. minor*. The suitability of species from the same family as hosts for root hemiparasites has been related to their growth rate (Hautier *et al*. 2010; Moncalvillo *et al*. 2025). However, in the current study, *S. minor* was a much poorer host than *L. perenne*, although its growth was much stronger. This suggests that among unrelated species, factors other than their size and rate of growth are more important components of host quality.

### Survival and growth of the parasites in phase 2

Prior parasitism of the hosts by the root hemiparasite *R. alectorolophus* in phase 1 affected both the survival and the biomass of the hemiparasites *R. alectorolophus* and *M. arvense* in phase 2. The survival of the parasites was lower if the host plants had been parasitized previously. This effect was largely consistent across the two parasite species and most pronounced with the host *L. perenne*, which had been suppressed most strongly by parasitism during phase 1. The lower survival of the parasites on previously parasitized hosts indicates a reduced probability of successful attachment to the hosts, due to an increase in host defences in response to the first infection. Attacks by parasitic plants can induce similar defence responses as those produced by herbivores and pathogens (Runyon *et al*. 2010; Tjiurutue *et al*. 2016; Clarke *et al*. 2019; Jhu & Sinha 2022). These responses include increases in the levels of jasmonic acid, salicylic acid and other phytohormones, which may result in the creation of physical barriers to penetration by the haustoria through hypersensitive‐like responses or the lignification of cell walls (Albert *et al*. 2021; Mutuku *et al*. 2021; Jhu & Sinha 2022).

A possible alternative explanation of the lower survival of the parasites on previously parasitized hosts is that due to their reduced size, these hosts were less likely to provide the parasites with sufficient resources. However, adjusting for host size at the start of phase 2 did not change the results qualitatively, suggesting that the reduced parasite survival was indeed due to failure to attach to the host.

Hemiparasite seedlings that were unable to attach to the host were likely outcompeted by the much larger hosts and died. Especially in high‐productivity ecosystems, unattached hemiparasites often do not survive, even if they are facultative parasites and can grow without a host (Fibich *et al*. 2010; Těšitel *et al*. 2015). There was little evidence that the effect of prior parasitism on the survival of the parasite differed between *R. alectorolophus* and *M. arvense*, indicating that prior parasitism unlocks general defence mechanisms that are effective against several related parasitic species. General immune responses induced by herbivores and pathogens are widespread in plants (Reimer‐Michalski & Conrath 2016), where exposure to a certain herbivore or pathogen may result in increased defence against a second attack by such enemies (Conrath *et al*. 2006; Howe & Jander 2008; Doughari 2015; Albanova *et al*. 2023). However, in addition to the general effects of prior infection on the survival of the two hemiparasites, there were specific interactions between the individual parasite and host species. When grown with *T. repens*, survival of *M. arvense* was much higher than that of *R. alectorolophus*, but this was not the case with the other hosts.

Other studies investigating the effects of prior parasitism of the hosts on subsequent parasitic plant performance (Seel & Press 1996; Puustinen & Salonen 1999; Tjiurutue *et al*. 2016) did not study survival. Prior parasitism of tomatoes by the shoot parasite *Cuscuta* and subsequent removal of the parasite slightly reduced the speed of attachment of *Cuscuta* during a second infection, but not its overall success (Tjiurutue *et al*. 2016). The authors suggested various mechanisms that could be responsible for this effect: a different scent profile of the hosts, scents produced by the dead *Cuscuta* or induced defence mechanisms.

While prior parasitism of the host increased parasite mortality, the surviving parasites were much larger than those grown with control hosts. The induced defence which led to increased mortality may have selected parasite seedlings that were most able to overcome host defences and extract resources. Selection of the fittest parasite individuals may thus have been responsible for the higher mean biomass of parasites that grew with hosts that had previously been parasitized. Moreover, as shown by their reduced growth, parasitization during phase 1 weakened the host plants, which may have allowed stronger exploitation of the hosts by the parasites, as stressed hosts are generally less able to resist parasite or pathogen infection (Gehring & Whitham 1992; Pennings & Callaway 2002). However, parasite growth in phase 2 was not influenced by host size at the start of phase 2, but apart from reducing host growth, parasitism may affect various physiological processes of the hosts (Salonen & Lammi 2001; Watling & Press 2001; Spallek *et al*. 2017), which may have allowed increased growth of the parasites. A positive effect of prior parasitism on parasite growth was also found for *R. minor* when it was grown with *Poa alpina* for a second year (Seel & Press 1996), but it was attributed to changes in the allocation patterns of the hosts due to parasitism (no production of plantlets, instead more and larger leaves).

The lower survival of hemiparasite seedlings and the stronger growth of the survivors as a consequence of prior parasitism of the hosts in the present experiment resembles the response of hemiparasites to increased nutrient availability and productivity (Van Hulst *et al*. 1987; Joshi *et al*. 2000; Těšitel *et al*. 2013) but are due to a different mechanism. Increased host biomass results in stronger shading by the hosts and higher mortality because hemiparasite seedlings are very sensitive to shading (Těšitel *et al*. 2011). However, the surviving parasites may then profit from a greater amount of resources (nutrients and water, but also some carbon; Těšitel *et al*. 2011) extracted from their larger hosts, but also from the higher nutrient levels available to their own root systems. Similarly, *R. minor* seedlings planted closer to a host suffered higher mortality than those planted further away (Keith *et al*. 2004), but the surviving parasites grew larger and had a higher fecundity. This was attributed to the negative effects of stronger shading near the host on seedling survival, on the one hand, and the positive effects of a longer period of heterotrophic growth and better access to the host root system closer to the host, on the other hand. In contrast to the effects of increased productivity and proximity to the host in these studies, we found increased parasite mortality and performance in response to prior parasitism of the host, although the hosts were actually smaller. Thus, the negative effects on parasite survival were not due to increased shading and the positive effects on parasite size were not due to higher resource availability from a larger host.

Depending on whether host responses that lead to (partial) resistance against parasitic plants act before or after attachment, these mechanisms have been classified as pre‐attachment or post‐attachment resistance (Jhu & Sinha 2022). Parasitism by *R. alectorolophus* induced pre‐attachment defences that reduced the probability of successful attachment of the parasites to the hosts, but did not increase post‐attachment resistance. The stronger growth of the parasites with previously parasitized hosts may even reflect the costs of the induced pre‐attachment defence mechanisms, if those resulted in less efficient post‐attachment defences.

### Effects of parasitism on the hosts during phase 2

Negative effects of parasitism in phase 1 on the biomass of *L. perenne* and *S. minor* were still noticeable at the time of harvest at the end of phase 2 three months later. Three months of growth without a parasite were not sufficient for them to recover from parasitism. Interestingly, these negative effects could only partially be explained by the reduction in host biomass during phase 1, suggesting that the reduced biomass was not due to simple carry‐over effects from phase 1. Moreover, it was also not due to changes in the pattern of allocation to roots and shoots of the hosts, as parasitism in phase 1 had no effect on the root mass fraction (RMF) of the hosts at the end of the experiment. In other studies, parasitism commonly increased the RMF of the hosts (see review in Matthies 2017; Korell *et al*. 2020), due to a loss of below‐ground resources, which stimulates root growth. The negative effects of parasitism by *R. minor* and *R. serotinus* on the biomass of perennial hosts were still visible a year later, which may have important implications for plant communities consisting of perennial host plants (Seel & Press 1996; Puustinen & Salonen 1999).

*T. repens* differed from the other two host species in that its biomass at the end of phase 2 was no longer negatively influenced by prior parasitism during phase 1 and also not by parasitism in phase 2, except in the case of a second infection by *R. alectorolophus*. *Trifolium repens*, and some other legumes have been found to be rather tolerant of parasitism, supporting large parasites without suffering strong damage (Matthies 2017, 2021; Sandner & Matthies 2018). This tolerance of parasitism has been attributed to their strong growth but is not universal (Moncalvillo *et al*. 2025). In the current study, *T. repens* grew very large and produced more biomass than the other two host species, which may have prevented a negative effect of parasitism in phase 2. However, the particularly strong reduction of the growth of *T. repens* when infected for a second time by *R. alectorolophus* shows that the tolerance of *T. repens* of parasitism is limited, and that after an infection, the species becomes very sensitive to further parasitism. This sensitivity could not be explained by the reduction of biomass due to parasitism in phase 1 and was also not due to changes in the pattern of allocation to roots and shoots (RMF).

Generally, the negative effects of parasitism by *R. alectorolophus* on naive hosts that had no prior experience of parasitism were weaker in phase 2 than in phase 1. In phase 2, the hosts were older and had accumulated more biomass. Large hosts may act as stronger competitors for light and resources taken up by the roots (Matthies 1995, 2017) but may also be more beneficial to hemiparasites by providing more water, nutrients and carbon (Seel & Press 1996; Li *et al*. 2015; Cirocco *et al*. 2020). Host age is known to influence plant defence responses to parasitic plants (Runyon *et al*. 2010; Moncalvillo & Matthies 2023), insects and pathogens (Elger *et al*. 2009; Panter & Jones 2009). Older hosts were less strongly suppressed by root parasitic plants than younger hosts in several studies (Cechin & Press 1993; Seel & Press 1996; Li *et al*. 2015; Cirocco *et al*. 2020; Moncalvillo & Matthies 2023). Young host plants may lack effective defences against parasitism when compared to older individuals (Runyon *et al*. 2010).

For *M. arvense*, *S. minor* was a more beneficial host than the other two host species. Survival of the parasite was more than 80%, and its biomass was highest. *S. minor* was also found to be a very good host for *M. arvense* by Matthies (2017). None of the host species were damaged by *M. arvense*, which grew less strongly than *R. alectorolophus*.

In conclusion, root hemiparasites grown with hosts that had been parasitized previously had a lower probability of survival but a higher mean biomass than parasites grown with inexperienced hosts. This indicates that parasitism induces defence mechanisms in the host that hinder future parasite attachment (pre‐attachment resistance). However, once a parasite has successfully attached, the induced defence does not appear to be effective (no increased post‐attachment resistance). In the field, individual host plants are often infected by several parasitic plant individuals, and infection by a single parasite individual may therefore impede later attachment of other parasite individuals. This could be in particular a problem for other root hemiparasites like *Euphrasia* spp. and *Odontites* spp., which often grow at the same sites as *Rhinanthus* spp., but germinate much later. *Rhinanthus* spp. already germinate in late autumn (Sandner & Matthies 2017), and their root systems develop over winter and may already form haustoria with host roots. Once *Euphrasia* and *Odontites* germinate in spring and start to make contact with host roots, host defences against root hemiparasites may thus already have been induced.

We did not investigate whether the resistance induced by hemiparasite attack of the hosts in spring will still be present in the next growing season. However, because parasite infection has long‐term negative effects on the growth of perennial hosts, it may also facilitate the growth of hemiparasites in the following years. Moreover, the increased resistance to further infection after parasitization by a root hemiparasite suggests that the classic pathways involved in the defence of plants against herbivores and pathogens are also activated by the infection of plants by root hemiparasites, which may result in increased defence in subsequent interactions with pathogens or herbivores that are long‐lasting (Henry *et al*. 2013). For example, when maize was infected by the functional holoparasite *Striga hermonthica*, consumption of its leaves by larvae of the moth *Chilo partellus* was reduced (Mohamed *et al*. 2007). Such cascading effects (Chaudron *et al*. 2021) could potentially alter community dynamics. However, our results also indicate that while there were general effects of parasitization on the defence of hosts, there were also specific interactions between individual pairs of hemiparasite and host species.

## AUTHOR CONTRIBUTIONS

SW and DM designed the study. SW performed the experiments. SW and DM analysed the data and wrote the manuscript. All authors approved the final manuscript.

## Supporting information


**Fig. S1.** Box plots showing the biomass of the parasite *Rhinanthus alectorolophus* grown with three different host species (*Lolium perenne, Sanguisorba minor, Trifolium repens*).
**Fig. S2.** Box plots showing the estimated biomass of the host species at the end of phase 1 of the experiment when grown with and without *Rhinanthus alectorolophus*.
**Fig. S3.** Box plots showing the biomass of the parasites *Rhinanthus alectorolophus* and *Melampyrum arvense* at the end of phase 2 when grown with three different hosts which had been parasitized or not by *R. alectorolophus* in phase 1.
**Fig. S4.** Box plots showing the biomass of the host plants at the end of phase 2. The host plants had been parasitized or not by *Rhinanthus alectorolophus* in phase 1 and were grown without a parasite, with the parasite *R. alectorolophus* or with the parasite *Melampyrum arvense* in phase 2.
