# Supplementary material for: Induced defence by a root hemiparasite increases host plant resistance against future infection
Source: Plant Biol (Stuttg). 2026 Feb 1;28(4):1279–88. doi: 10.1111/plb.70187 (PMC13175950; doi:10.1111/plb.70187)
Supplement: Supplementary file 1 — Fig. S1. Box plots showing the biomass of the parasite Rhinanthus alectorolophus grown with three different host species (Lolium perenne, Sanguisorba minor, Trifolium repens). Fig. S2. Box plots showing the estimated biomass of the host species at the end of phase 1 of the experiment when grown with and without Rhinanthus alectorolophus. Fig. S3. Box plots showing the biomass of the parasites Rhinanthus alectorolophus and Melampyrum arvense at the end of phase 2 when grown with three different hosts which had been parasitized or not by R. alectorolophus in phase 1. Fig. S4. Box plots showing the biomass of the host plants at the end of phase 2. The host plants had been parasitized or not by Rhinanthus alectorolophus in phase 1 and were grown without a parasite, with the parasite R. alectorolophus or with the parasite Melampyrum arvense in phase 2. [file PLB-28-1279-s001.docx]

Supporting information S1 to Wanke and Matthies


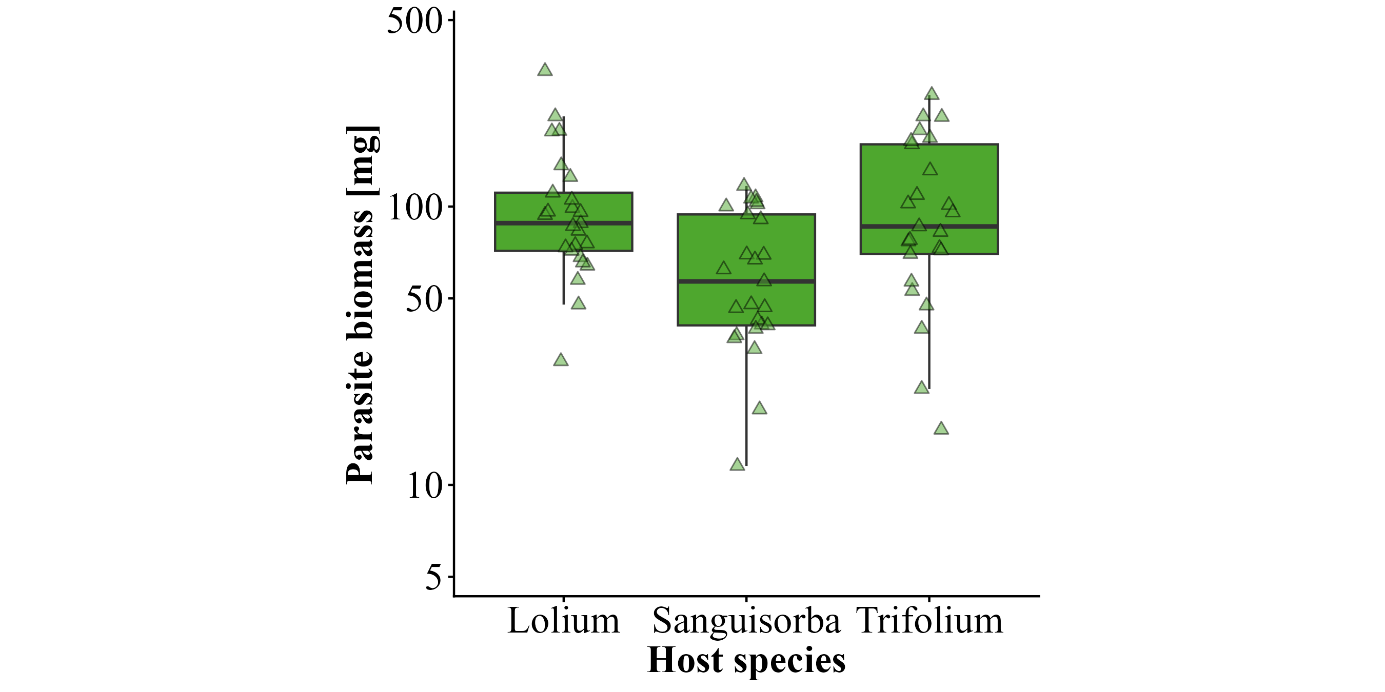


Fig. S1: Box plots showing the biomass of the parasite *R. alectorolophus* grown with three different host species (*L. perenne, S. minor, T. repens*).


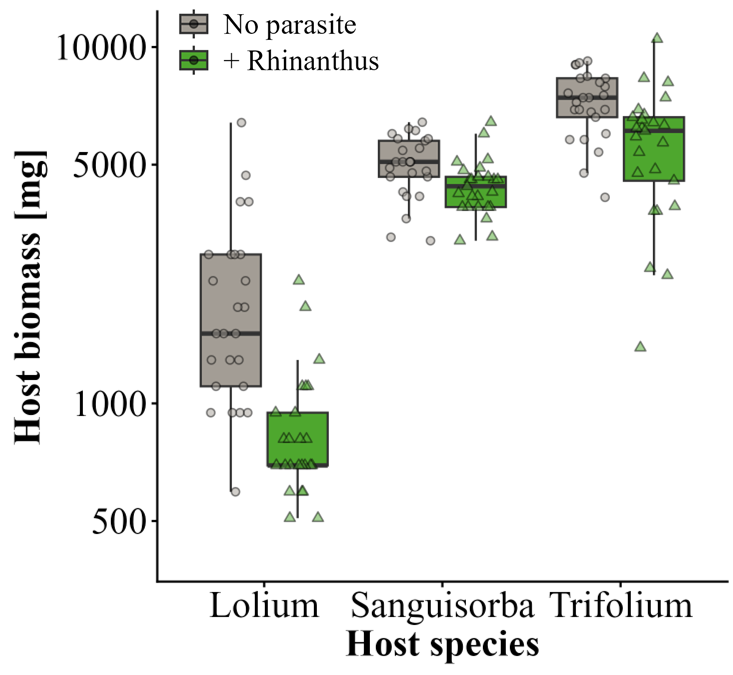


Fig. S2: Box plots showing the estimated biomass of the host species at the end of phase 1 of the experiment when grown with and without *R. alectorolophus*.


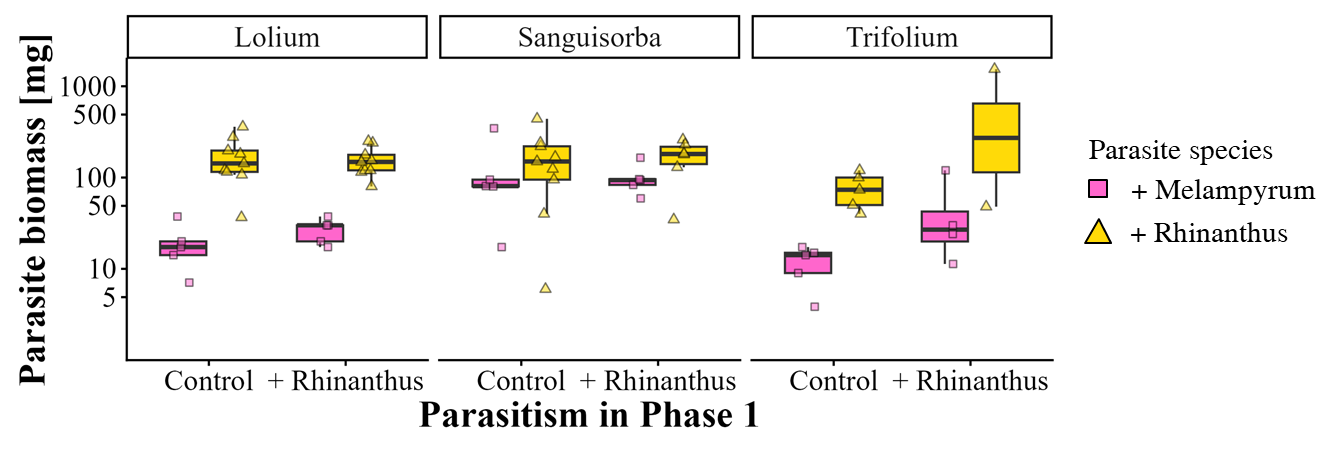


Fig. S3: Box plots showing the biomass of the parasites *Rhinanthus* *alectorolophus* and *Melampyrum* *arvense* at the end of phase 2 when grown with three different hosts which had been parasitised or not by *R. alectorolophus* in phase 1.


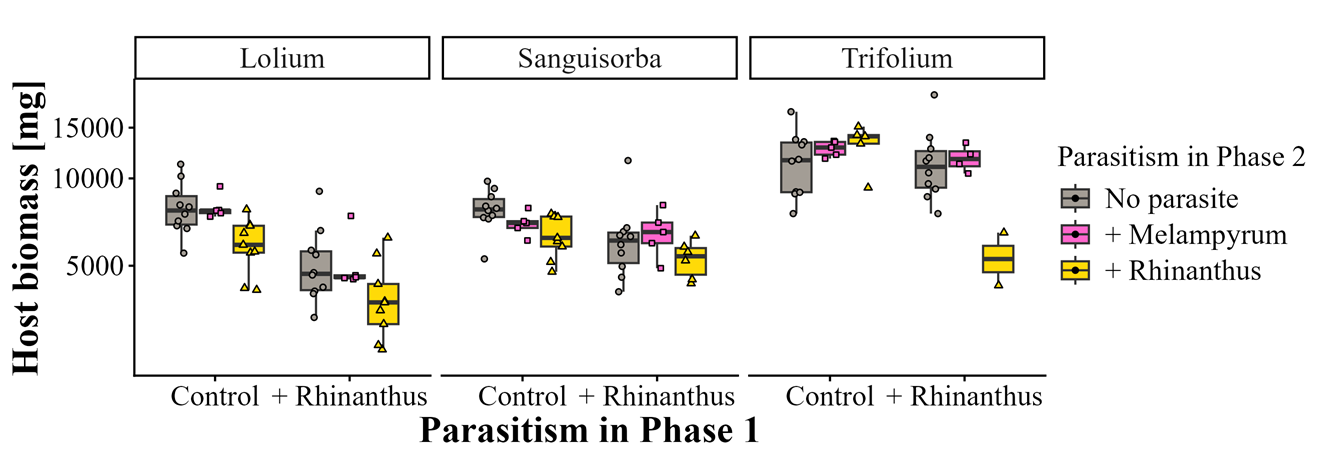


Fig. S4: Box plots showing the biomass of the host plants at the end of phase 2. The host plants had been parasitised or not by *R. alectorolophus* in phase 1 and were grown without a parasite, with the parasite *R. alectorolophus* or with the parasite *Melampyrum* *arvense* in phase 2.
